# Professional identity formation amongst peer-mentors in a research-based mentoring programme

**DOI:** 10.1186/s12909-023-04718-y

**Published:** 2023-10-24

**Authors:** Lalit Kumar Radha Krishna, Anushka Pisupati, Kelly Jia Hui Teo, Mac Yu Kai Teo, Chrystie Wan Ning Quek, Keith Zi Yuan Chua, Vaishnavi Venktaramana, Vijayprasanth Raveendran, Harpreet Singh, Sabine Lauren Wong Chyi Hui, Victoria Wen Wei Ng, Ong Yun Ting, Eleanor Kei Ying Loh, Ting Ting Yeoh, Jasmine Lerk Juan Owyong, Eng Koon Ong, Gillian Li Gek Phua, Ruaraidh Hill, Stephen Mason, Simon Yew Kuang Ong

**Affiliations:** 1 Yong Loo Lin School of Medicine, National University of Singapore, NUHS Tower Block, 1E Kent Ridge Road, Singapore, 119228 Singapore; 2 Division of Supportive and Palliative Care, National Cancer Centre Singapore, 30 Hospital Boulevard, Singapore, 168583 Singapore; 3 Division of Cancer Education, National Cancer Centre Singapore, 30 Hospital Boulevard, Singapore, 168583 Singapore; 4 Duke-NUS Medical School, 8 College Road, Singapore, 169857 Singapore; 5 Lien Centre for Palliative Care, Duke-NUS Medical School, Singapore, 169857 Singapore; 6 Health Data Science, Institute of Population Health, University of Liverpool, Brownlow Street, Liverpool, L69 3GF UK; 7 Palliative Care Institute, University of Liverpool, 200 London Road, Liverpool, L3 9TA UK; 8 The Palliative Care Centre for Excellence in Research and Education (PalC), Dover Park Hospice, 10 Jln Tan Tock Seng, Singapore, 308436 Singapore; 9 Centre for Biomedical Ethics, National University of Singapore, 10 Medical Dr, Singapore, 117597 Singapore; 10 Division of Oncology Pharmacy, National Cancer Centre Singapore, 30 Hospital Boulevard, Singapore, 168583 Singapore; 11 Assisi Hospice, 832 Thomson Rd, Singapore, 574627 Singapore; 12 Office of Medical Humanities, SingHealth Medicine Academic Clinical Programme, Singapore, Singapore; 13 Division of Medical Oncology, National Cancer Centre Singapore, 30 Hospital Boulevard, Singapore, 168583 Singapore

**Keywords:** Community of practice, Medical students, Medicine, Mentoring, Professional identity formation, Physicians, Peer mentoring, Socialization

## Abstract

**Background:**

Mentoring plays a pivotal yet poorly understood role in shaping a physician’s professional identity formation (PIF) or how they see, feel and act as professionals. New theories posit that mentoring nurtures PIF by functioning as a community of practice through its structured approach and its support of a socialisation process made possible by its assessment-directed personalized support. To test this theory and reshape the design, employ and support of mentoring programs, we evaluate peer-mentor experiences within the Palliative Medicine Initiative’s structured research mentoring program.

**Methods:**

Semi-structured interviews with peer mentors under the Palliative Medicine Initiative (PMI) at National Cancer Centre Singapore were conducted and triangulated against mentoring diaries to capture longitudinal data of their PMI experiences. The Systematic Evidence-Based Approach (SEBA) was adopted to enhance the trustworthiness of the data. SEBA employed concurrent content and thematic analysis of the data to ensure a comprehensive review. The Jigsaw Perspective merged complementary themes and categories identified to create themes/categories. The themes/categories were compared with prevailing studies on mentoring in the Funnelling Process to reaffirm their accuracy.

**Results:**

Twelve peer-mentors participated in the interviews and eight peer-mentors completed the mentoring diaries. The domains identified were community of practice and identity work.

**Conclusions:**

The PMI’s structured mentoring program functions as a community of practice supporting the socialisation process which shapes the peer-mentor’s belief system. Guided by a structured mentoring approach, stage-based assessments, and longitudinal mentoring and peer support, peer-mentors enhance their detection and evaluation of threats to their regnant belief system and adapt their self-concepts of identity and personhood to suit their context. These insights will help structure and support mentoring programs as they nurture PIF beyond Palliative Medicine.

## Background

Medical education is tasked with shaping the Professional Identity Formation (PIF) of medical students and physicians (henceforth clinicians), or how they think, feel and act as professionals as they navigate their medical practice [1, 2]. Extant literature [3–10] foregrounds two critical ingredients for the successful nurturing of PIF: the socialisation process and the Community of Practice (CoP) [3]. However, current understanding of these mechanisms remains rudimentary [11].

New insights into mentoring practice at the Palliative Medicine Initiative (PMI), a structured research mentoring program at the National Cancer Centre Singapore (NCCS), may offer a better perspective into the development of professional identities in this aspect of medical education. Indeed, mentoring is a recognised means of developing PIF in medical education. However, before we evaluate PMI experiences, its role in nurturing PIF and its links with CoP and the Socialisation Process, we must foreground our approach with explanations of the terms and theories we will use.

### The Palliative Medicine Initiative (PMI)

To begin, the PMI employs a combined novice-, peer- and e-mentoring approach (henceforth CNEP) [12, 13] that sees graduating PMI mentees nominated, recruited, trained, and mentored by senior mentors to support new PMI mentees. Having completed at least one PMI mentored research project and tutored on how to mentor, assess and provide feedback to peers and new mentees, the peer-mentors are re-orientated to the mentoring approach and their roles and responsibilities. Provided with a choice of projects to work on from systematic reviews in ethics, professionalism, communication, PIF, mentoring, thanatology, wellbeing, and reflective practice to qualitative interviews on palliative care, PIF and mentoring, peer-mentors often discuss these projects with the senior mentors involved, ascertaining the alignment of their expectations to work together. Peer-mentors are then provided with access to robust communication channels with the two senior mentors overseeing the project. At this stage, peer-mentors are reacquainted with the PMI’s norms, skills, motivations, and attitudes (henceforth *desired characteristics);* learning objectives, goals, timelines, professional standards, codes of conduct, roles, responsibilities, expectations, implicit norms, culture, artifacts, sociocultural norms and expectations and legal requirements (henceforth *codes of conduct*). They are also reintroduced to the current education approaches; the program’s value, support and assessment systems; the settings and stages of training, as well as the formal curriculum (henceforth *host organization related facets*).

Following their regular bi-weekly online meetings vis-à-vis ad-hoc and informal meetings, peer-mentors make an entry into their mentoring diaries to document their experiences. These entries are privy to only specific members of the administrative staff to safeguard the anonymity and privacy of the peer-mentors whilst ensuring that timely and appropriate support can be provided if any mentoring, mental health, and physical problems are detected.

**Fig. 1:**
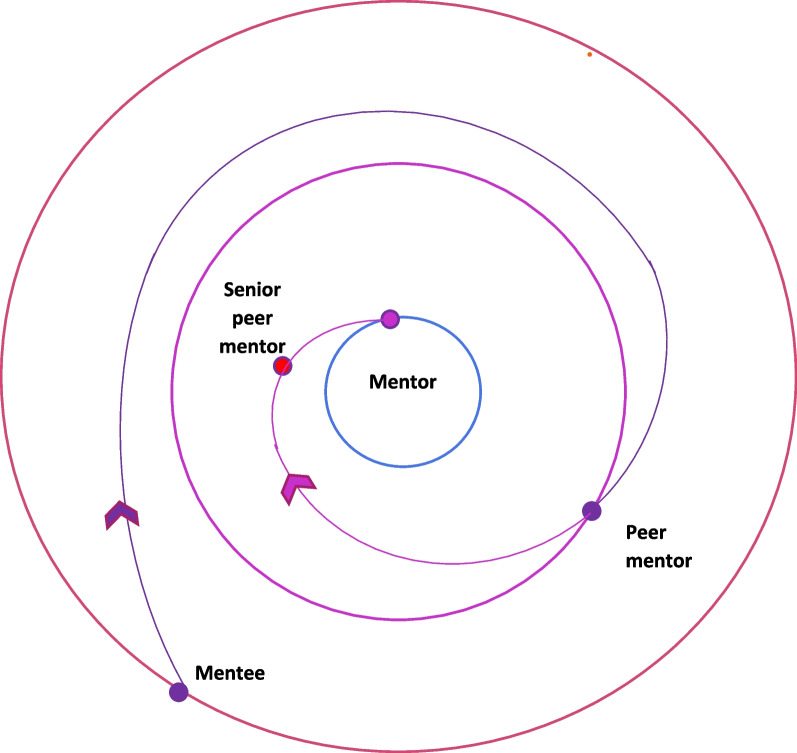
Planned spiral course for peer-mentors in the PMI

The PMI also establishes a spiral curriculum for peer-mentors that sees them regularly revisit key training skills and competencies along the training trajectory (Fig. 1). This spiral curriculum is designed on the PMI’s well-established mentoring stages [9]. These include the matching; initial meeting; data gathering and analysis; manuscript writing and submission; and post manuscript submission stages [9]. Each mentoring stage delves deeper into the knowledge, skills, attitudes and experiences of the previous stage. It also brings a new set of competencies to be attained. Moving from one stage to the other demands these competencies be met, creating natural ‘competency-based assessment points’ at transitions from one mentoring stage to the other [9]. These competencies must be met if a peer-mentor is to guide mentees from one stage to the next. The mentoring stages also map out the mentoring trajectory characterising the gradual inculcation of complex skills, knowledge, and competency in communications, relationships, learning, socialisation, collaborations, networking skills, reflective practice, medical humanism, and professionalism that will be employed to guide mentees [2, 11]. The peer-mentor’s mentoring trajectory follows that of the mentee and maps the peer-mentor’s progress in the PMI. Movement along this mentoring trajectory from the periphery of the PMI program towards more significant roles takes a spiral course representing the revisiting of knowledge, skills, and attitudes and achieving the new competencies for each mentoring stage.

These roles are represented by three concentric rings. The centre ring represents the role of a senior mentor whilst the middle and outermost ring denote the roles of the peer-mentor and mentee respectively in the CoP. Progress from one role to the next reflects achievement of the requisite skills and competencies, alongside concurrent changes in perspectives, belief systems, decision making and actions. Shepherding this developmental process is personalised, timely, appropriate, longitudinal, and often holistic mentoring support drawn from the mentoring umbrella, guided by mentoring assessments and provided by the two trained mentors. It is this combination of personalised mentoring support framed around the PMI’s structured approach and the mentoring course mapped out by the mentoring framework that facilitates the shift away from individual self-interests amongst mentees to furthering the interests of the PMI program that practices ‘paying it forward’. Facilitating this spiral course between the circles is a combination of experiential learning [14], graduated mentored immersion in the PMI culture and practice, graded autonomy and allocated responsibility [15, 16], structured revisiting of skills and knowledge to build competence and a developing sense of community, moral reasoning, and reflective judgment [5, 17–23].

The clearly defined trajectory and expectations set out provide a unique opportunity to study the longitudinal influence of mentoring on the PIF of peer-mentors [8–10, 13]. Indeed, ‘exit’ interviews of peer-mentors, along with their feedback and mentoring diaries, allude to changes in the peer-mentor’s values, beliefs, and principles (henceforth belief systems) and their thinking, conduct and practice. It is these findings that have inspired this study into peer-mentor experiences.

In particular, there are two features that are imperative to our study. The first is the mentoring umbrella [2–4, 10, 24] which sees an assessment-driven, context- specific, individualised mix of role modelling, networking, coaching, supervision, apprenticeship and traditional concepts of mentoring provided throughout the mentoring program that supports immersive learning and reflective practice. The second is the mentoring structure that includes the physical boundaries of the mentoring program, its curated mentoring environment that actively shapes the hidden and informal curricula [7], and stage-based mentoring [25] within a formal mentoring program overseen by the host organization [26]. The mentoring structure also includes clearly articulated *codes of conduct*, *desired characteristics* and *host organization related facets* [27–30]; mentored immersion [31] that nurtures experiential learning and builds personalised mentoring relationships; mapped mentoring trajectory [13]; stage-specific competency assessments [9]; longitudinal mentoring support [32]; mentor training programs [10]; and program assessment protocols [26]. Collectively, the mentoring structure guides the mentoring trajectory, assessment, support and program oversight.

### The Socialisation Process

The mentoring umbrella plays a key role in supporting the Socialisation Process, or the internalisation of “*the characteristics, values, and norms of the medical profession… resulting in an individual thinking, acting and feeling like a physician”* [1]. Here, the mentoring umbrella [24, 32, 33], together with longitudinal mentoring support and interactions with peers and mentees over time within the confines of the PMI’s codes of conduct and culture, facilitate the Socialisation Process to shape the peer-mentor’s internalisation of programmatic, speciality-specific, organizational, ethical, legal, professional, socio-culturally relevant values, beliefs, principles and mentoring insights, reflections, experiences, and knowledge.

### Community of practice

The Socialisation Process is, in turn, dependent on the presence of an organized, supportive sense of community provided by the PMI through its curated mentoring environment and mentoring structure. This likens the PMI to a Community of Practice (CoP). Indeed, the PMI’s mentoring structure does meet the key features of a CoP as “*a persistent, sustaining social network of individuals who share and develop an overlapping knowledge base, set of beliefs, values and history and* experiences *focused on a common practice and/or enterprise”* [34].

With its shared sense of identity, structure, culture and mentoring support, the PMI provides peer-mentors with a holistic and personalised means of inculcating new beliefs, values, principles, insights, and experiences into regnant belief systems within the confines set by the mentoring structure [6, 35, 36]. Together, the features of CoP and socialisation buttress the PIF of peer-mentors as they detect and adjudge the gravity of new beliefs, values, principles, and experiences upon their current belief systems and ensure their response remains within the confines of accepted standards. To advance a more accurate reflection of peer-mentor experiences in the PMI, we adopt the lens of the Krishna-Pisupati Model of Professional Identity Formation (KPM) and the Ring Theory of Personhood (RToP).

### The Krishna-Pisupati model of professional identity formation and the ring theory of personhood (RToP)

**Fig. 2:**
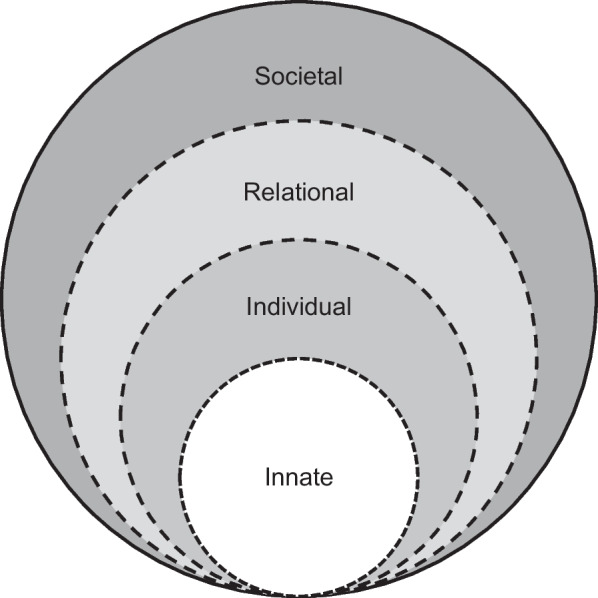
The ring theory of personhood

The RToP builds on the notion that shifts in a clinician’s belief systems change how they see themselves as persons and as professionals. This allows the RToP to map changes in individual belief systems in the clinician’s Innate, Individual, Relational and Societal identities and capture corresponding changes in the Innate, Individual, Relational and Societal rings of personhood (Fig. 2).

The Innate Identity is derived from the peer-mentor’s regnant spiritual, religious, theist, moral and ethical belief systems within the Innate Ring. The Individual Ring’s belief system revolves around the peer-mentor’s beliefs, values and principles surrounding conscious function and informs the peer-mentor’s thoughts, conduct, biases, narratives, personality, and decision-making processes, thus shaping the peer-mentor’s Individual Identity. The Relational Identity is born of a belief system governing those relationships that the peer-mentor determines to be personal and important. The Societal Identity is shaped by regnant societal, cultural, religious, professional, and legal roles and expectations, and belief systems which inform their interactions with colleagues and acquaintances.

The Individual Identity also manifests in the peer-mentor’s overall identity, balancing the influences of the Innate, Relational and Social Identities. This balancing process shapes and is shaped by the beliefs systems within each domain of personhood. When there is consistency between the beliefs, values, and principles being introduced and the current belief system within the ring or rings of the RToP, there is ‘resonance’. ‘Synchrony’ occurs when current values, beliefs and principles within the ring or rings of the RToP are reprioritised to better fit with the practice beliefs, values, and principles. In contrast, inconsistencies between new and prevailing belief systems lead to ‘dissonance’. Dissonance within one ring of the RToP precipitates ‘disharmony’ whilst ‘dyssynchrony’ manifests from dissonance between rings.

**Fig. 3:**
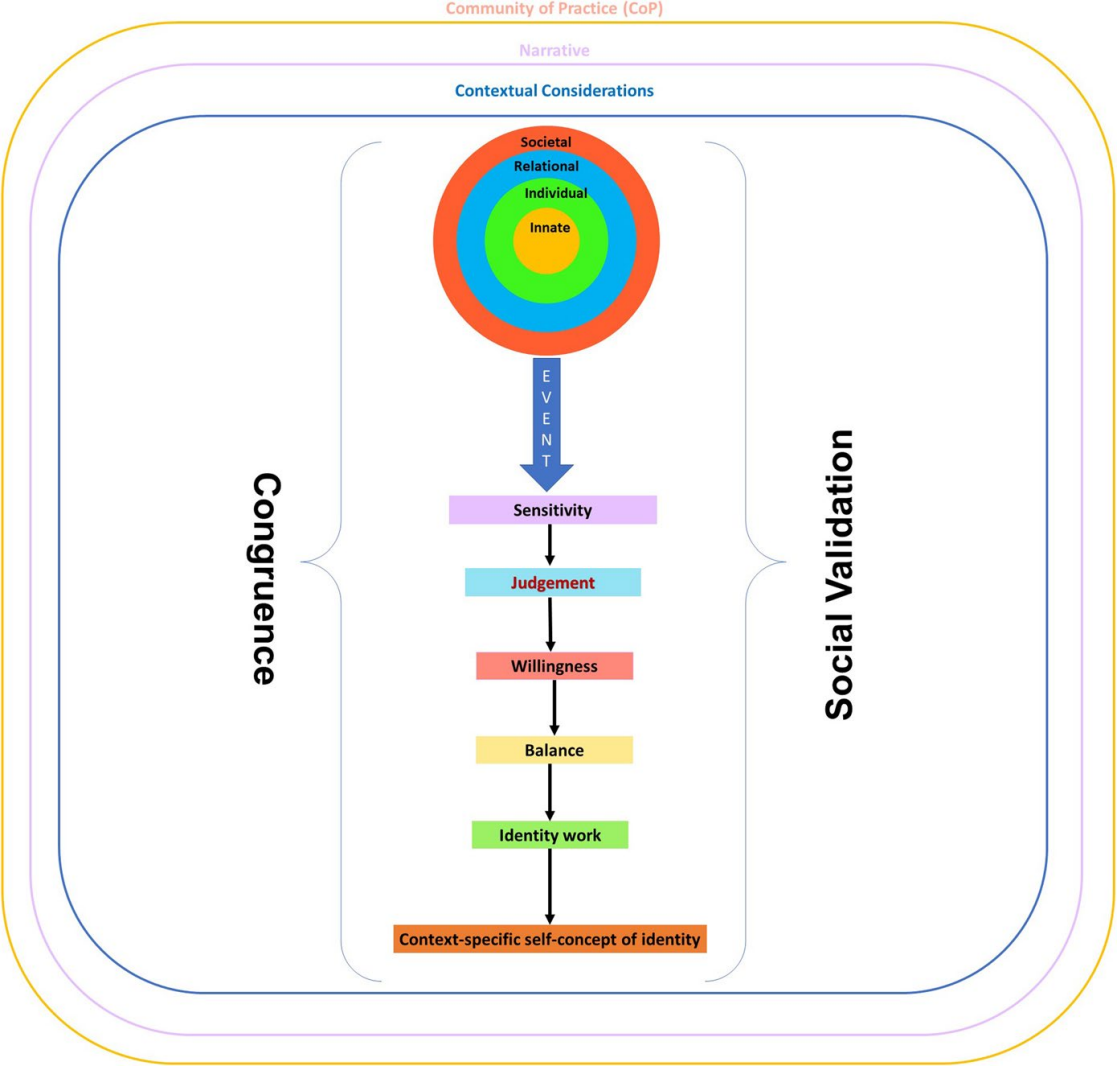
The Krishna-Pisupati model for professional identity formation

The KPM explores the effects of resonance, synchrony, dyssynchrony and disharmony (henceforth event) on the belief system and PIF (Fig. 3). The KPM suggests that detection of an ‘event’ is determined by the mentee’s ‘sensitivity’ whilst their ‘judgment’ displays the significance of the ‘event’ upon their current belief system. The KPM also captures the notion of ‘willingness’ to address the ‘event’ and the clinician’s ability, experience and opportunity to ‘balance’ possible adaptations in response to the ‘event’ with regnant practical, clinical, personal, sociocultural, professional, academic and organizational considerations culminating in the creation of a context-specific self-concept of identity. The KPM works best within a ‘closed’ or structured mentoring program and a well-surveilled environment to proffer a means of studying the longitudinal effects of a consistent mentoring approach.

It is within these conditions where the PMI functions as a CoP supporting the Socialisation Process and that changes in the peer-mentor’s belief system may be captured by the KPM and RToP that we pose the primary research question, “How does peer-mentoring in a structured mentoring program impact PIF?”. In providing a comprehensive perspective of peer-mentoring experiences in the PMI, we proffer the secondary research questions, “What features of the CoPs and the socialisation process are present in the PMI peer-mentoring experience?” and “What impact does mentoring have on Professional Identity Formation?”.

### Methodology

Acknowledging peer-mentoring as a sociocultural construct shaped by individual, psycho-social, academic, professional, clinical, research and environmental considerations, we adopt a Constructivist perspective and a Relativist lens. Accordingly, we adopt a qualitative approach to study the lived experiences of peer-mentors in the PMI. To capture a longitudinal and holistic perspective of peer-mentoring experiences, we employ a semi-structured interview questionnaire and peer-mentoring diaries. We analyzed the data from the semi-structured interviews and diaries using Krishna’s Systematic Evidence Based Approach (SEBA).

#### Stage 1 of SEBA: Expert advice

**Fig. 4:**
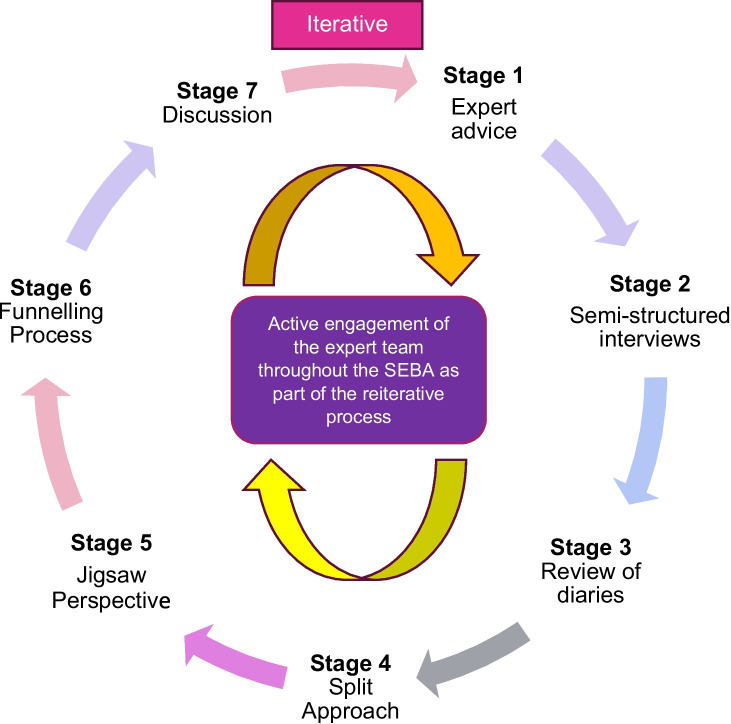
The SSR in SEBA process

In advancing a balanced, accountable and reproducible study and analysis, an expert team consisting of a librarian from the National University of Singapore’s (NUS) Yong Loo Lin School of Medicine (YLLSoM) and local educational experts and clinicians at YLLSoM, National Cancer Centre Singapore, Palliative Care Institute Liverpool, and Duke-NUS Medical School navigated the stages of SEBA (Fig. 4).

#### Stage 2 of SEBA: Semi-structured interviews

Eligible participants comprised of peer-mentors in the PMI who had completed mentoring programs as mentees and had been subsequently selected, trained and completed PMI mentoring programs as peer-mentors. Purposive sampling was conducted and email invitations containing a participant information sheet and consent form were sent out. The invitations stressed the participant’s rights to privacy and anonymity, as well as their right to withdraw from the study at any point without prejudice. All participants provided written and verbal informed consent.

Individual semi-structured interviews were arranged with each peer-mentor upon return of the endorsed consent forms. These 30–45-min audio-recorded interviews conducted over the Zoom video conferencing platform took place in quiet offices that ensured privacy to facilitate in-depth exploration of personal belief, experiences and practices. The interviews were carried out between February and May 2021 by experienced and trained interviewers, AP and CQWL. As non-clinicians with neither previous interactions nor dependent relationships with the participants, the trained interviewers sent out the email invitations and arranged the meetings. This enhanced the participant anonymity from the research and expert teams. Audio recordings were transcribed verbatim using the NVivo 12 Software, anonymized and their integrity verified.

### Ethical considerations

Ethics approval (reference number: 202010–00084 and 202103–00057) was obtained from the Singhealth Combined Institutional Review Board. Informed written and oral consent was obtained from all participants.

#### Stage 3 of SEBA: Review of mentoring diaries

Peer-mentoring diaries were hosted on Google Forms and were completed by all mentees and peer-mentors in the PMI on an ad-hoc basis between March to December 2021. Mentoring diaries were anonymized by independent research team members not involved in the PMI or the semi-structured interviews for analysis.

#### Stage 4 of SEBA. Split approach

Three independent teams, each guided by a senior trained PMI mentor, carried out the analysis of the anonymized data. The first and second teams employed Braun and Clarke [37]’s approach to thematic analysis and Hsieh and Shannon [38]’s approach to Directed Content Analysis respectively to analyze the transcripts of the semi-structured interviews. The second team drew categories for the content analysis from Sarraf-Yazdi et al. [11]’s review “A scoping review of professional identity formation in undergraduate medical education”. The third team carried out thematic and content analysis of the peer-mentoring diaries.

At independent and regular online discussions, Sandelowski and Barroso [39]’s approach to ‘negotiated consensual validation’ was used to reach consensus on the codes identified. As the coding process was part of mentor led training and subject to frequent expert team input, Kappa inter-reliability scores were not evaluated.

#### Stage 5 of SEBA: Jigsaw perspective

This process combined overlapping themes and categories to create themes/categories.

#### Stage 6 of SEBA: Funneling process

The themes/categories from the mentoring diaries and interviews were combined and funneled to create domains that frame the discussion in Stage 8 [40].

## Results

Twelve peer-mentors participated in the study interviews, and a further eight peer-mentors completed the peer-mentoring diaries. Table 1 depicts the demographic information of the participating peer-mentors, including the number of projects undertaken over their course of time in the PMI.

**Table 1:** Participant demographic

**Study Interviews**			
**Peer-Mentors (P)**	**Student Year**	**No. of projects undertaken**	**Duration involved (years)**
P1	PGY3	3	3
P2	PGY1	9	2
P3	PGY1	5	1
P4	Y4	4	1
P5	Y4	4	2
P6	Y4	6	3
P7	Y5	6	1
P8	Y5	2	1
P9	Y4	2	2
P10	Y2	5	2
P11	Y4	2	1
P12	Y4	2	1
**Mentoring Diaries**			
**Peer Mentors’ Diaries (PD)**	**Student Year**	**No. of projects**	**Duration involved (years)**
PD1	PGY3	4	3
PD2	Y4	3	1
PD3	Y4	6	2
PD4	Y2	12	2
PD5	Y2	12	2.5
PD6	Y4	6	4
PD7	Y4	3	2
PD8	Y3	5	2

Independent analysis of the interviews with 12 peer-mentors and 8 peer-mentor diaries revealed two domains—the PMI as a Community of Practice, and Identity Formation.

### Domain 1. PMI as a community of practice (CoP)

The PMI displays features of a CoP described by existing literature [41–46]. Firstly, the PMI exhibits a robust mentoring structure that creates the physical boundaries of the mentoring program, its curated mentoring environment [7], and stage-based mentoring trajectory [25] within a formal mentoring program overseen by the host organization [26]. These facets establish the boundaries of the PMI’s CoP.

The PMI’s strong sense of community, welcoming environment, culture of ‘paying it forward’, and shared belief systems create the CoP’s culture whilst the mentoring umbrella-based support strengthens a peer mentor’s core values of responsibility, teamwork, empathy, respect, integrity and commitment, alongside their shared belief system and identity.

Progress along the PMI’s mentoring trajectory guided by the mentoring umbrella-based support within the clear boundaries of the PMI’s structure and nurturing mentoring environment supports the Socialisation Process. This helps peer-mentors integrate the PMI’s belief system and indeed apply them in the program. This bolsters personal and professional development as peer mentors are encouraged and challenged to adopt new roles and responsibilities.

In toto, the PMI’s sense of community, structured approach, support mechanisms and fostering of personal and professional development solidify its position as a CoP. Table 2 expounds on each key characteristic below.

**Table 2:** CoP features identified in the PMI

Theme: PMI as a Community of Practice (CoP)	
**CoP Features**	**Quotes**
**Subtheme 1. A Sense of Community** A sense of community was created by a shared sense of identity, values, culture, and approach. Common values instilled amongst peer-mentors include kindness, patience, responsibility, empathy, sensitivity, humility, service orientation, reducing hierarchy, teamwork, honesty, commitment, approachability, integrity, respect, effective and open communications and ‘paying it forward’. This shared culture also extended beyond that seen within the medical schools and local hospital practice	
**Shared Identity**	“This idea of being generous or paying it forward or giving back to people got reinforced during my process as a near-peer” (P4)
**Shared Approach**	“Like in a music ensemble…each of them have their own scores, each of them go home and rehearse their own parts. And when it comes together, it creates beautiful music.” (P1)
**Shared Values**	“I stayed up on call to review the paper and put in my comments here and there even though there was no way I was getting into the publication. But there are certain things you do without expectations… Do good without expecting anything in return.” (P1)
**Shared Culture**	“It's a nice principle that [the mentor] goes by and I think definitely would like to apply that in both this PMI and also in areas beyond research. (P7)
	“It's kind of weird that I've not really heard about this culture outside of the program, mainly learned about it from the program.” (P6)
**Variety of Goals**	Peer mentors joined the PMI for a variety of reasons. These included the desire to ‘pay it forward’(P1) and to improve teaching (PD P1, P3, P4, P5, P9), writing (PD5), leadership and time management skills. (P9, P12)
**Welcoming Environment**	“Thankful to be in this programme where I am given good opportunities not just to grow but also to guide others and learn together” (PD8)
	“My mentors show amazing sense of patience and are really caring about what others are going through.” (P10)
	“Whenever he meets us he would ask us how are we? What are we doing right now? I think that's nice of him to show interest to all of us as people.” (P11)
**Subtheme 2. Structured Approach** Whilst often considered part of the mentoring environment, a pivotal aspect of the PMI experience is its **structured approach**. This included a structured yet personalized recruitment process that considered the peer-mentor’s narrative and goals, the establishment of clear goals and expectations, and the commencement of a guided mentoring process that was personalized and flexible to meet changing conditions and the different stages of the mentoring process	
**Personalized Recruitment**	“The first meeting was to talk about how things were looking, what was the context of the work, and what are the next steps, and if I was open to suggestions [on research, projects, working styles and feedback in general]” (P9)
**Personalized Goals**	“Goal settings is one of the things that we started off with… in terms of what I plan to take out of research, and the PMI.” (P5)
	“Understanding the motivations behind joining PMI, and the research project…allows tailor[ing]…expectations and goals accordingly.” (P1)
**Clear Expectations**	“In the PMI.. everything was very clear at the start, what the role is and how much work you have to put in.” (P11)
**Guided and Staged Process**	“We are allocated roles from the start, and moving on gradually from mentee stage to a mentor stage…from how you code, its effects to how you write your own paper.” (P8)
	“There is a step-by-step guide to starting the paper, the search strategy, how to convert the search strategy to other databases, then the TIAB sieve and introduction to Endnote…all laid out there.” (P10)
	“There is someone to guide me through each stage of the research project.” (P11)
**Flexible and Adaptive**	“If it gets stressful because of our commitment, studies and stuff, he's very flexible and always tells us that studies comes fast and to tell him a deadline that we're good with.” (P12)
**Subtheme 3. Support Mechanism** There were a number of **support mechanisms** employed. The nature of the feedback in the PMI also influenced the relationships, program, and success. They were deemed constructive, timely, personalized and often encouraging	
**Role Modelling**	“[The mentor] showed…how a doctor should be in society…and that influences my thinking of how a well-rounded doctor should be in society.” (P7)
	“[Facing study and work problems]…I reached out to him and he was really nice about it. And very understanding. He was really the embodiment of like the spirit of the PMI…I guess I just want to be like him also.” (P5)
**Holistic and Accessible Support**	“If you ask her questions, she will reply very, very quickly, and she would be very detailed in her response as well. So, I think that really helps to make things clear.” (P9)
	“They are approachable, so I feel braver to ask questions about the project and advice on life.” (PD6)
	“My mentor has been very supportive of the struggles I am experiencing in personal life, but also has been very patient with my inexperience and blunders made along the way.” (PD7)
**Career Support**	“We speak about other stuff [too]…such as school, like help with content in medical school, career guidance, advice, as well as personal life.” (P1)
**Feedback**	“When I made that really big mistake. I felt really, really bad.. But my Senior Mentor was very understanding.. it made me want to be the same in the future.” (P4)
	“My senior mentor usually reverts to me with comments or proposed changes within a day for the drafts I submit. I think it shows his dedication to his work.” (PD1)
	“Think along the way, when peers and seniors mentors give very constructive feedbacks and encouraging remarks for the work and the improvement, then I think that sort of builds confidence.” (P3)
**Subtheme 4. Personal and Professional Development** Peer-mentors were **challenged** to take on more responsibilities and roles as they gained confidence, knowledge, skills, and experience. Reflecting on their PMI experiences, peer-mentors reported changes in their personal values and choices, practice styles, career choices, and perspectives towards **self-care**	
**Challenge and Growth**	“So, I went through the same process for a couple of papers…then along the way…my Senior Mentor and the peer-mentors said “you should try guide new students into how to ease into this stages of this process of writing a paper.” (P3)
	“My confidence built after completing one project as a mentee and as a team player. Subsequently then I was first author and had mentees under me for the project with the Senior mentor.” (P9)
	“It's made me less materialistic and [focus] more on fields of work where I can provide a listening ear to people, and generally provide more holistic care because that's what my mentor did for me.” (P1)
	“I learned that things that will give me more fulfilment in a job would go beyond the superficial enjoyment, but also include fields of work where I will be able to listen to people more and understand people more. Like for example, in family medicine, we provide holistic care.” (P2)
**Self-Care**	“I feel a deeper obligation to mentor and raise juniors to become thoughtful and mentally healthy doctors through PIF.” (PD3)

### Domain 2. professional identity formation

Peer mentors exhibit features of PIF in their efforts to attend to resonance, synchrony, dyssynchrony and disharmony. Resonance, for instance, is apparent when peer mentors find that the values, beliefs, principles, and goals introduced in the PMI echo that of their prevailing belief systems. Resonance motivates continued engagement in the PMI and sees peer mentors reshape and reprioritise their regnant values, beliefs and principles to reflect synchrony and inspire action. Here, peer mentors show personal growth in taking on new endeavours not just for their individual self-development, but to find meaning in serving the wider community. However, peer-mentors also encounter obstacles in their PMI journey, alerted by their ‘sensitivity’ to instances of disharmony and dyssynchrony. Disharmony manifests when peer mentors reveal a mismatch between their prevailing skillsets and new responsibilities whilst managing ‘difficult’ mentees who miss deadlines or produce inadequate work heightens dyssynchrony.

Dyssynchrony, disharmony, resonance, and synchrony may arise as an acute ‘event’, such as the rejection of a white-listed journal submission (P10) or dealing with ‘difficult’ mentees (P11). The precipitating ‘event’ may also be a slow process simmering or persistent issues such as dealing with a lack of ‘confidence’ (P5) and self-belief (P2), contending with changing perspectives of oneself and roles (P2), balancing competing demands on time and maintaining a work-life balance (P3).

How and if dyssynchrony, disharmony, resonance, and synchrony are to be addressed is determined by the peer-mentor’s ‘judgement’ that accords attention, significance and urgency to the issue. Often, ‘judgement’ is perceived as a matter of goal setting, prioritising tasks, time and roles and managing expectations. Upon determining the magnitude of the situation, the peer mentor’s motivation to make adaptations to their self-concepts of identity pivots on their ‘willingness’. This is depicted as part of the peer-mentor’s responsibility (P6), accountability (P11), goals (P7) and aspirations (P4).

Peer mentors also exhibit ‘balance’ in determining the changes to be carried out and prioritised in making adjustments to their self-concepts of identity. The process of balancing is also impacted by the speed in which events occur, the peer-mentor’s ‘sensitivity’, ‘judgment’ and ‘willingness’ and if there are further changes in the peer-mentor’s circumstances. This then culminates in peer mentors practicing ‘identity work’ that sees the integration of new work practices, values, beliefs and principles, ‘sensitivity’, ‘judgment’, ‘willingness’ and ‘balance’ to create context-specific self-concept of identity.

Table 3 brings together the features of PIF identified in the PMI in more detail.

**Table 3:** PIF features identified in the PMI

Theme: Features of Professional Identity Formation (PIF) in the PMI	
**Subtheme 1. Sensitivity** ‘Sensitivity’ refers to awareness of the presence of dyssynchrony, disharmony, resonance, and synchrony	
**Resonance**	Resonance became evident when regnant belief systems were consistent with the new values, beliefs and principles being inculcated. Resonance motivates continued engagement
	“I find it [peer-mentoring role] meaningful, so I will pursue further studies or further development in research, as it's aligned with my goals of helping people” (P3)
	“This experience as a peer-mentor in PMI has helped to force me to actively practice what I learn at church and my spiritual classes” (P9)
**Synchrony**	Reshaping and reprioritising regnant values, beliefs and principles inspired action
	**Innate Ring:** “Karma. I’m not very religious…nor spiritual but…I believe what goes around comes around. …it really taught me not to be so transactional.” (P1)
	**Individual Ring:** “I'm proud of myself for actually taking this step. I think the old me wouldn't really have bothered with all these things…I want to be a helping hand [to PMI mentees].” (P5)
	**Relational Ring:** “I realised that it's possible to be friends, to be close to them and get to know them without losing their respect for you. This is something that I learnt.” (P9)
	**Societal Ring:** “I became more focused on other people, I find more meaning and serving people, rather than just focusing on myself and my own development” (P3)
**Dyssynchrony**	**Between the Individual and Relational Rings:** Relating to managing a ‘difficult’ mentee. “She didn't reply very much. And when she did work, it was not very good…so the work was done by the other members of team but she was also my batch-mate so it was a little bit difficult in terms of how to communicate [this].” (P3)
	**Between the Individual and Societal Ring:** “While I was losing enthusiasm, I had to continue motivating my juniors, who were losing enthusiasm as well.” (P1)
**Disharmony**	Peer-mentors experienced disharmony within their Societal ring when called upon to take on new responsibilities that they felt they were not equipped for. They also faced disharmony when they had to make decisions that, in their opinion, involved additional work and stress for the team:
	“So in this particular instance, I do not think I was ready. Purely because research is not one of my strong point…I think it would affect my confidence in mentoring others, because the last thing I want would be to bring them down the wrong path.” (P1)
	“I worry as a peer-mentor that I am not being a good mentor or leader. I do feel bad about giving work and rushing deadlines.” (PD2)
	“For my relationship with mentees, I feel like I am lacking in some ways as a leader and sometimes I feel scared that I’m causing unnecessary work and stress for them.” (PD6)
**Subtheme 2. Judgement** ‘Judgement’ determines if the ‘event’ detected warrants attention, its significance and urgency	
Influences	Goals (P1),
	prioritising tasks (P9),
	time (P12)
	roles (10)
	and or managing expectations (P7)
**Subtheme 3. Willingness** The peer-mentor’s ‘willingness’ determines whether the peer-mentor is motivated make adaptations to their self-concepts of identity	
Motivations arise from	Sense of responsibility (P6),
	accountability (P11),
	goals (P7)
	and aspirations (P4)
**Subtheme 4. Balance** Making adjustments to create a context-specific self-concept of identity may involve multiple changes to belief systems and different aspects of self-concepts of identity and personhood. Determining which changes are to be carried out, prioritised and the extent that these changes would be made hinges on the notion of ‘balance’	
Influenced by	speed in which events occur,
	the peer-mentor’s sensitivity’,
	‘judgment’
	and ‘willingness’
	and if there are further changes in the peer-mentor’s circumstances
**Subtheme 5. Identity work** The integration of new work practices, values, beliefs and principles, ‘sensitivity’, ‘judgment’, ‘willingness’ and ‘balance’ influences identity work or the changes required to create context-specific self-concept of identity. ‘Identity work’ involves a combination of role modelling, guidance and mentoring by senior peer-mentors (P12) and mentors (P10)	
Role modelled	adopt the practice, approach, style and personal characteristics that they admire or feel will be helpful to advancing their goals or needs (P6)
Personal characteristics	being approachable (P6), sensitive (P7), understanding (P2), caring (P3), kind (P5), empathetic and humble (P9), adaptable (P10) and being accountable (P1)
Mentoring related practices	attention to detail (P8), collaborative (P8), communication (P4) and feedback styles (P5), social and personal awareness of the mentee’s needs (P1), maintaining professional boundaries (P1), mentoring approaches (P8), building a ‘pay it forward’ (P4) and reflective (P3) mindset, maintaining mentee motivations (P6), facilitating an effective work-life balance (P2); and nurturing independence (P11) and resilience (P12) amongst mentees
Influencing factors	PMI’s structure (P11); the tone and culture of the program (P6); management approaches (P6); a map of the course of the mentoring process (P3); clearly established goals and codes of practice (P10); alignment of expectation (P2); accessible lines of communication (P4); timely (P12), accessible (P4), welcoming (P9), personalized (P11), longitudinal and personalized guidance (P8), mentoring (P1), and leadership support (P9); a nurturing environment (P5); regular meetings with mentors (P7); and personalized (P12) and respectful (P7) mentoring relationships

#### Stage 7 of SEBA: Discussion

**Fig. 5:**
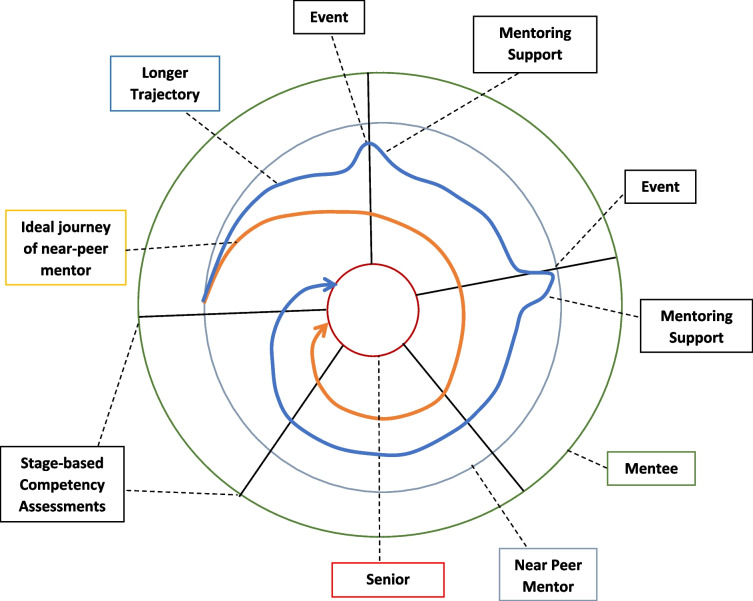
The trajectory of peer-mentors in CoPs

This study reveals that a structured program like the PMI does act as a CoP supporting the socialisation process capable of nurturing PIF amongst peer-mentors. Through the lens of the KPM and RToP, it is possible to appreciate the impact of resonance, synchrony, dyssynchrony, and/or disharmony on belief systems and its effects upon the conduct and practice of peer-mentors as they progress along the mentoring trajectory set out (Fig. 5).

Dependent on the peer-mentor’s individual experience, capabilities, goals, abilities, and availabilities, it is common that the course taken by new peer-mentors deviates from the ideal (blue line). Here, the role of the mentoring umbrella helps them stay on course and as close to the ideal trajectory. However, as the data suggests, changes in the peer-mentor’s personal, psycho-social, financial, clinical, research, academic, practice and/or psycho-emotional situation may lead to a significant ‘event’. Here, the presence of regular stage-based assessments help direct further personalised and appropriate support to peer-mentors. If effective, these interventions bring the peer mentoring trajectory back towards the ideal trajectory. It is posited that with these interventions, peer-mentors will eventually achieve their goals.

Our data also reveals a further reason for deviations from the ideal mentoring trajectory. Peer-mentors appear to be simultaneously involved to varying degrees in different roles in concurrent projects. Whilst concurrent participation in multiple mentored programs within the PMI may impact progress in their primary project, evidence of multiple frequently overlapping programs within the PMI casts the PMI as a collection of projects, each functioning as a CoP. This implications of a “complex landscape of different communities of practice” invites the idea of the PMI being a Landscape of Practice (LoP) [27, 46–56] and raises a number of considerations.

For peer-mentors involved in several concurrent projects (multi-membership), often in different capacities in a LoP, ‘events’ have wider connotations and are subject to wider influences. Here, subtle differences even amongst projects built on a common PMI belief system may be problematic due to possible differences in the support and advice provided by various mentors. This underscores the need for longitudinal and holistic evaluation of peer-mentor’s progress.

**Fig. 6:**
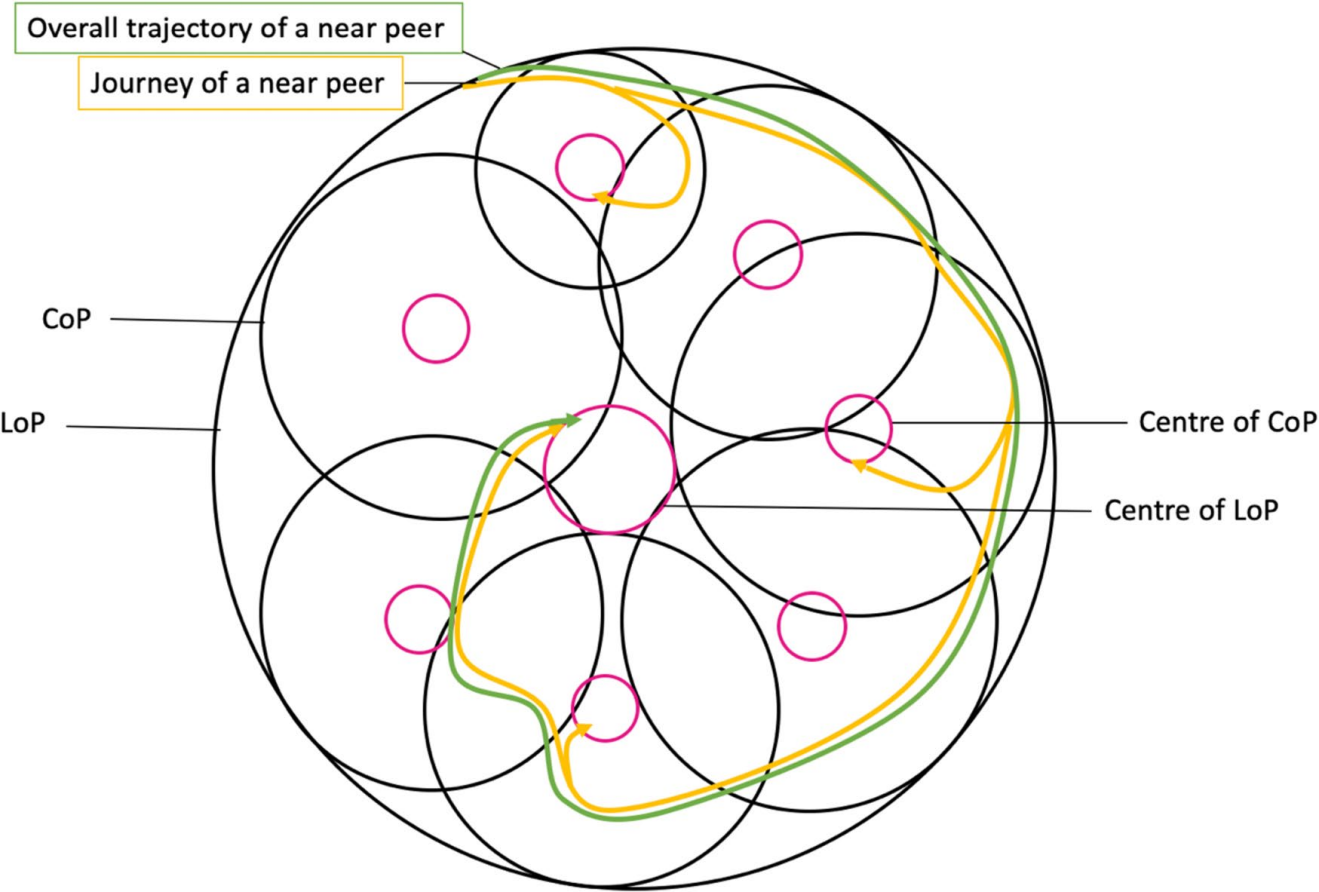
Community of practice and landscape of practice

On the surface, being a LoP underlines the importance of the PMI, ensuring consistency in the culture, goals, belief systems and structure within projects/CoPs. It also stresses the importance of ensuring that PMI mentors are effectively trained and supported, and that there is alignment of expectations; timely, stage-based and appropriate assessments; oversight; and seamless support across the PMI. On a deeper level, however, it does shift the manner in which progress is viewed within the PMI. Indeed, it may be suggested that progress within multiple PMI projects may be viewed as the peer-mentor’s overall progress towards refining their PIF (Fig. 6).

### Updating the Krishna-Pisupati’s theoretical model of professional identity formation

**Fig. 7:**
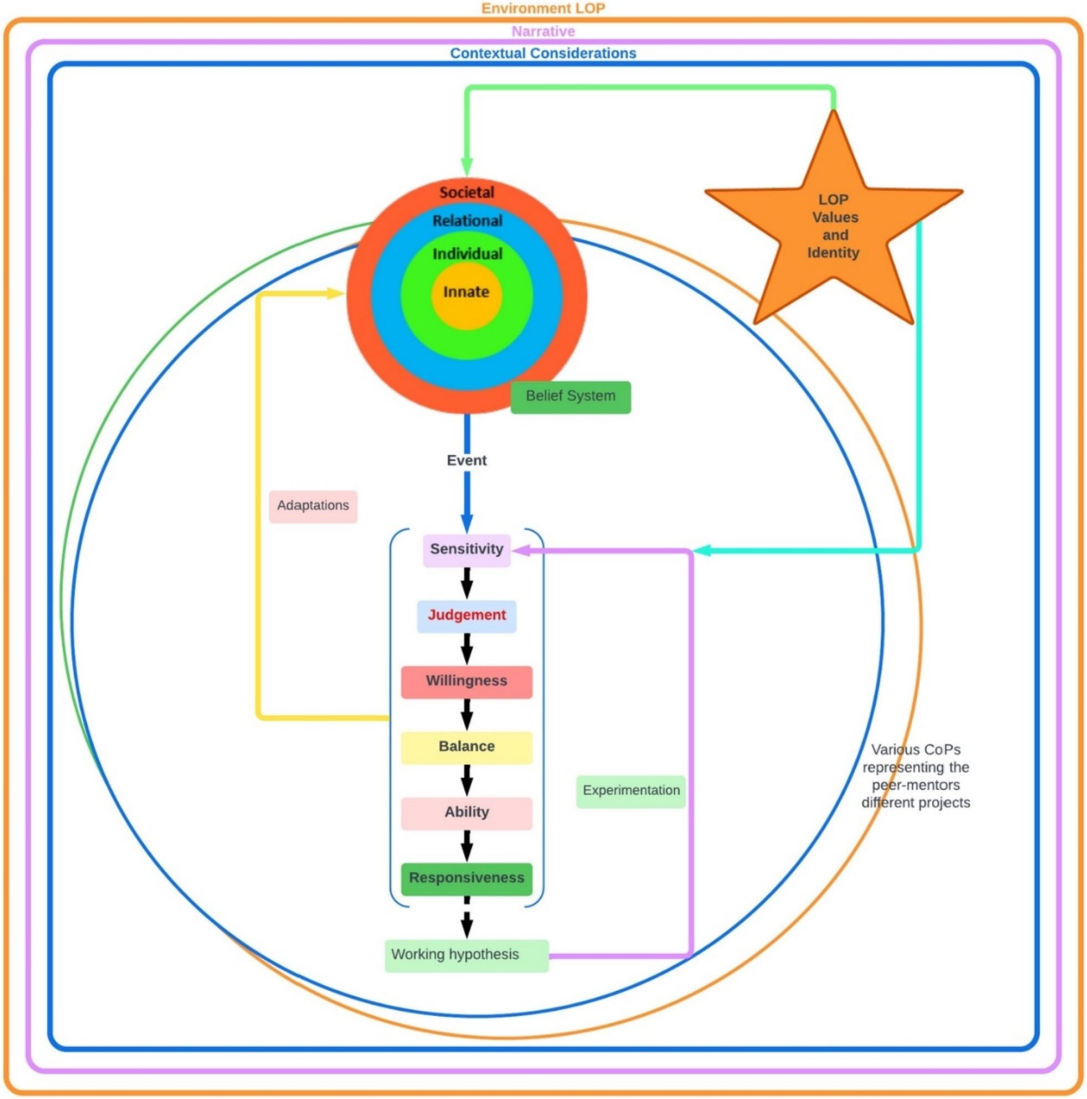
The updated Krishna-Pisupati framework for PIF in mentoring

With the KPM increasingly proposed as an evidence-based approach to better understand PIF in mentoring and potentially in medical education, ensuring that the KPM is clinically relevant is critical. The notion of the PMI being a LoP thus inspires a change in the manner that we view Krishna-Pisupati’s theoretical model (Fig. 7).

Situated within the LoP which also captures the wider contextual and environmental considerations, the advanced KPM is still focused upon the PIF of an individual. As a result, there remains due acknowledgment of the influence of the individual’s narratives. ‘Sensitivity’, ‘judgment’, ‘willingness’, and ‘balance’ leading up to the creation of working hypothesis for a context-specific identity that can straddle all the PMI projects now also considers the peer-mentor’s ability and responsiveness, as well as the notion that events need not be singular nor from one source, but from multiple sources. Events may also exhibit differing levels of importance and exigency. This underscores the importance of continued guidance and support of peer-mentors as they rank ‘events’ in terms of importance and significance.

Experimenting with the working hypothesis or ‘adapted’ professional identity and belief systems is guided by available support, culture and structure of the CoPs and the LoP, as well as regnant codes of practice, boundaries, expectations, roles and responsibilities set out by the LoP and CoPs.

### Limitations

Although insightful, this qualitative approach built upon the RToP has not been validated and is subject to bias interpretations by the authors. Moreover, although supplemented by mentoring diaries, the use of interviews as the primary source of data in this study remains as ‘snapshots’ or retrospective accounts that are susceptible to recall bias. There are also limitations due to the small sample size and the limit of the depth of the data collected.

Whilst the categories drawn from directed content analysis emphasised the features of CoPs in the PMI and complemented the themes identified in Identity Formation, the Split Approach and Funnelling Processes are time and resource-heavy and could threaten the sustainability of the study. Similarly, use of independent team analysis and the Split Approach may not have fully attenuated the risk of bias.

## Conclusion

This study suggests that a structured mentoring program can shape professional identity in a consistent manner as long as there is due consideration for the needs of the peer-mentor and the influence of environmental factors. The impact of environmental factors is multiplied when multi-membership is present and underlines the need for portfolio use that will not only assess their competency, but their PIF and promote the use of reflective practice and mentoring diaries. This then must be an area for future study.

Similarly, whilst this study will be of particular interest to program designers, host organizations and senior mentors, it does reiterate the need for host organizations to ensure careful stakeholder selection, training, assessment, and support; effective alignment of expectations; longitudinal support and assessment of the mentoring, communication, assessment, and oversight mechanisms within the program; and careful curation of the mentoring culture and structure. This underlines the need for effective program evaluations. These gaps in appraisals ought to be the focus of future studies as we plan to extend the PMI beyond Palliative Medicine.

## Data Availability

All data generated or analysed during this study are included in this published article.

## References

[1] Cruess RL, Cruess SR, Steinert Y (2018). Medicine as a Community of Practice: Implications for Medical Education. Acad Med.

[2] Venktaramana V, Ong YT, Yeo JW, Pisupati A, Krishna LKR (2023). Understanding mentoring relationships between mentees, peer and senior mentors. BMC Med Educ.

[3] Teo KJH, Teo MYK, Pisupati A, Ong RSR, Goh CK, Seah CHX (2022). Assessing professional identity formation (PIF) amongst medical students in Oncology and Palliative Medicine postings: a SEBA guided scoping review. BMC Palliat Care.

[4] Lim JY, Ong SYK, Ng CYH, Chan KLE, Wu S, So WZ (2023). A systematic scoping review of reflective writing in medical education. BMC Med Educ.

[5] Wald HS, Anthony D, Hutchinson TA, Liben S, Smilovitch M, Donato AA (2015). Professional identity formation in medical education for humanistic, resilient physicians: pedagogic strategies for bridging theory to practice. Acad Med.

[6] Chong JY, Ching AH, Renganathan Y, Lim WQ, Toh YP, Mason S (2020). Enhancing mentoring experiences through e-mentoring: a systematic scoping review of e-mentoring programs between 2000 and 2017. Adv Health Sci Educ Theory Pract.

[7] Hee JM, Yap HW, Ong ZX, Quek SQM, Toh YP, Mason S (2019). Understanding the Mentoring Environment Through Thematic Analysis of the Learning Environment in Medical Education: a Systematic Review. J Gen Intern Med.

[8] Krishna LKR, Tan LHE, Ong YT, Tay KT, Hee JM, Chiam M (2020). Enhancing Mentoring in Palliative Care: An Evidence Based Mentoring Framework. J Med Educ Curric Dev.

[9] Krishna LKR, Toh YP, Mason S, Kanesvaran R (2019). Mentoring stages: A study of undergraduate mentoring in palliative medicine in Singapore. PLoS ONE.

[10] Ong YT, Quek CWN, Pisupati A, Loh EKY, Venktaramana V, Chiam M (2022). Mentoring future mentors in undergraduate medical education. PLoS ONE.

[11] Sarraf-Yazdi S, Teo YN, How AEH, Teo YH, Goh S, Kow CS (2021). A Scoping Review of Professional Identity Formation in Undergraduate Medical Education. J Gen Intern Med.

[12] Lim SYS, Koh EYH, Tan BJX, Toh YP, Mason S, Krishna LKR (2020). Enhancing geriatric oncology training through a combination of novice mentoring and peer and near-peer mentoring: A thematic analysis ofmentoring in medicine between 2000 and 2017. J Geriatr Oncol.

[13] Krishna L, Tay KT, Yap HW, Koh ZYK, Ng YX, Ong YT (2020). Combined novice, near-peer, e-mentoring palliative medicine program: A mixed method study in Singapore. PLoS ONE.

[14] Cruess S, Cruess R (2012). Teaching professionalism - Why, What and How. Facts Views Vis Obgyn.

[15] Cruess R, Cruess S, Steinert Y (2016). Amending Miller's Pyramid to Include Professional Identity Formation. Acad Med.

[16] Sternszus R, Boudreau J, Cruess R, Cruess S, Macdonald M, Steinert Y (2020). Clinical Teachers' Perceptions of Their Role in Professional Identity Formation. Acad Med.

[17] Wald H, White J, Reis S, Esquibel A, Anthony D (2019). Grappling with complexity: Medical students’ reflective writings about challenging patient encounters as a window into professional identity formation. Med Teach.

[18] Wald HS (2011). Guiding Our Learners in Reflective Writing: A Practical Approach. Lit Med.

[19] Wald H, McFarland J, Markovina I (2019). Medical humanities in medical education and practice. Med Teach.

[20] Wald H (2020). Optimizing resilience and wellbeing for healthcare professions trainees and healthcare professionals during public health crises – Practical tips for an ‘integrative resilience’ approach. Med Teach.

[21] Wald H, Reis S, Monroe A, Borkan J (2010). The Loss of My Elderly Patient:’ Interactive reflective writing to support medical students’ rites of passage. Med Teach.

[22] Wald HS (2015). Professional identity (trans)formation in medical education: reflection, relationship, resilience. Acad Med.

[23] Wald H (2015). Refining a definition of reflection for the being as well as doing the work of a physician. Med Teach..

[24] Koh EYH, Koh KK, Renganathan Y, Krishna L (2023). Role modelling in professional identity formation: a systematic scoping review. BMC Med Educ.

[25] Goh S, Wong RSM, Quah ELY, Chua KZY, Lim WQ, Ng ADR (2022). Mentoring in palliative medicine in the time of covid-19: a systematic scoping review. BMC Med Educ.

[26] Chia EWY, Tay KT, Xiao S, Teo YH, Ong YT, Chiam M (2020). The Pivotal Role of Host Organizations in Enhancing Mentoring in Internal Medicine: A Scoping Review. J Med Educ Curric Dev.

[27] Wahab M, Ikbal M, Jingting W, Wesley L, Kanesvaran R, Krishna L. Creating Effective Interprofessional Mentoring Relationships in Palliative Care- Lessons from Medicine, Nursing, Surgery and Social Work. J Palliat Care Medi. 2016;06. 10.4172/2165-7386.1000290.

[28] Toh YP, Lam B (2017). Developing Palliative Care Physicians through Mentoring Relationships. Palliative Medicine &.

[29] Sng J, Pei Y, Toh YP, Peh TY, Neo Sh, Krishna L (2017). Mentoring relationships between senior physicians and junior doctors and/or medical students: A thematic review. Med Teach..

[30] Hee J, Toh YL, Yap HW, Toh YP, Kanesvaran R, Mason S (2020). The Development and Design of a Framework to Match Mentees and Mentors Through a Systematic Review andThematic Analysis of Mentoring Programs Between 2000 and 2015. Mentoring & Tutoring: Partnership in Learning.

[31] Tan YS, Teo SWA, Pei Y, Sng JH, Yap HW, Toh YP (2018). A framework for mentoring of medical students: thematic analysis of mentoring programmes between 2000 and 2015. Adv Health Sci Educ.

[32] Toh RQE, Koh KK, Lua JK, Wong RSM, Quah ELY, Panda A (2022). The role of mentoring, supervision, coaching, teaching and instruction on professional identity formation: a systematic scoping review. BMC Med Educ.

[33] Radha Krishna LK, Renganathan Y, Tay KT, Tan BJX, Chong JY, Ching AH (2019). Educational roles as a continuum of mentoring's role in medicine - a systematic review and thematic analysis of educational studies from 2000 to 2018. BMC Med Educ.

[34] Barab S (2003). Makinster J.

[35] Sawatsky AP, Santivasi WL, Nordhues HC, Vaa BE, Ratelle JT, Beckman TJ (2020). Autonomy and professional identity formation in residency training: A qualitative study. Med Educ.

[36] Sawatsky A, Nordhues H, Merry S, Bashir M, Hafferty F (2018). Transformative Learning and Professional Identity Formation During International Health Electives: A Qualitative Study Using Grounded Theory. Acad Med.

[37] Braun V, Clarke V (2006). Using thematic analysis in psychology. Qual Res Psychol.

[38] Hsieh H-F, Shannon SE (2005). Three Approaches to Qualitative Content Analysis. Qual Health Res.

[39] Sandelowski M, Barroso J (2006). Handbook for synthesizing qualitative research.

[40] Lingard L, Watling C (2016). It’s a Story, Not a Study: Writing an Effective Research Paper. Acad Med.

[41] Cruess RL, Cruess SR, Boudreau JD, Snell L, Steinert Y (2015). A schematic representation of the professional identity formation and socialization of medical students and residents: a guide for medical educators. Acad Med.

[42] de Carvalho-Filho MA, Tio RA, Steinert Y (2020). Twelve tips for implementing a community of practice for faculty development. Med Teach.

[43] Hean S, Anderson E, Bainbridge L, Clark PG, Craddock D, Doucet S (2013). IN-2-THEORY–Interprofessional theory, scholarship and collaboration: a community of practice. J Interprof Care.

[44] Sherbino J, Snell L, Dath D, Dojeiji S, Abbott C, Frank JR. A national clinician-educator program: a model of an effective community of practice. Medical education online. 2010;15(10.3402/meo.v15i0.5356). 10.3402/meo.v15i0.5356 PMC3000230 21151594

[45] Hägg-Martinell A, Hult H, Henriksson P, Kiessling A (2016). Community of practice and student interaction at an acute medical ward: An ethnographic study. Med Teach.

[46] Buckley H, Steinert Y, Regehr G, Nimmon L (2019). When I say … community of practice. Med Educ.

[47] Kuek JTY, Ngiam LXL, Kamal NHA, Chia JL, Chan NPX, Abdurrahman A (2020). The impact of caring for dying patients in intensive care units on a physician's personhood: a systematic scoping review. Philos Ethics Humanit Med.

[48] Wenger-Trayner E, Wenger-Trayner B (2015). Learning in a landscape of practice: A framework. Learning in landscapes of practice: Boundaries, identity, and knowledgeability in practice-based learning.

[49] Balmer DF, Rosenblatt S, Boyer D (2021). Navigating landscapes of practice: A longitudinal qualitative study of physicians in medical education. Med Educ.

[50] Sethi A, Ajjawi R, McAleer S, Schofield S (2017). Exploring the tensions of being and becoming a medical educator. BMC Med Educ.

[51] Hodson N (2020). Landscapes of practice in medical education. Med Educ.

[52] Wenger-Trayner E, McDermott R, Snyder W. Cultivating Communities of Practice: A Guide to Managing Knowledge; 2002.

[53] Bok C, Ng CH, Koh JWH, Ong ZH, Ghazali HZB, Tan LHE (2020). Interprofessional communication (IPC) for medical students: a scoping review. BMC Med Educ.

[54] Ng CH, Ong ZH, Koh JWH, Ang RZE, Tan LHS, Tay KT (2020). Enhancing Interprofessional Communications Training in Internal Medicine. Lessons Drawn From a Systematic Scoping Review From 2000 to 2018. J Contin Educ Health Prof..

[55] Ong ZH, Tan LHE, Ghazali HZB, Ong YT, Koh JWH, Ang RZE (2021). A Systematic Scoping Review on Pedagogical Strategies of Interprofessional Communication for Physicians in Emergency Medicine. J Med Educ Curric Dev.

[56] Tay KT, Tan XH, Tan LHE, Vythilingam D, Chin AMC, Loh V (2021). A systematic scoping review and thematic analysis of interprofessional mentoring in medicine from 2000 to 2019. J Interprof Care.

